# The Role of Sphingolipids and Sphingosine-1-phosphate—Sphingosine-1-phosphate-receptor Signaling in Psoriasis

**DOI:** 10.3390/cells12192352

**Published:** 2023-09-26

**Authors:** Kana Masuda-Kuroki, Shahrzad Alimohammadi, Anna Di Nardo

**Affiliations:** Department of Dermatology, School of Medicine, University of California San Diego, La Jolla, CA 92093, USA; kkuroki@health.ucsd.edu (K.M.-K.); shalimohammadi@health.ucsd.edu (S.A.)

**Keywords:** sphingolipid, ceramide, sphingosine-1-phosphate, sphingosine-1-phosphate receptor, psoriasis

## Abstract

Psoriasis is a long-lasting skin condition characterized by redness and thick silver scales on the skin’s surface. It involves various skin cells, including keratinocytes, dendritic cells, T lymphocytes, and neutrophils. The treatments for psoriasis range from topical to systemic therapies, but they only alleviate the symptoms and do not provide a fundamental cure. Moreover, systemic treatments have the disadvantage of suppressing the entire body’s immune system. Therefore, a new treatment strategy with minimal impact on the immune system is required. Recent studies have shown that sphingolipid metabolites, particularly ceramide and sphingosine-1-phosphate (S1P), play a significant role in psoriasis. Specific S1P–S1P-receptor (S1PR) signaling pathways have been identified as crucial to psoriasis inflammation. Based on these findings, S1PR modulators have been investigated and have been found to improve psoriasis inflammation. This review will discuss the metabolic pathways of sphingolipids, the individual functions of these metabolites, and their potential as a new therapeutic approach to psoriasis.

## 1. Introduction

Psoriasis is one of the common chronic skin disorders, and is characterized by elevated erythematous plaques with silver scales. Psoriasis has several forms: psoriasis vulgaris (plaque psoriasis), psoriatic arthritis, generalized pustular psoriasis, guttate psoriasis, and erythrodermic psoriasis, with psoriasis vulgaris representing the most significant percentage of patients [1,2]. Psoriasis generally occurs at a wide range of ages, from adolescence to middle age, and can also occur in children, but its prevalence depends on ethnicity [3]. The treatment approaches for psoriasis include topical steroids and vitamin D3, ultraviolet (UV) light therapy including narrow-band UVB and psoralen with UVA (PUVA), biologic agents, and oral Janus kinase (JAK) inhibitors [4,5]. However, all these cures are only targeted at reducing inflammation; thus, symptoms flare up when treatments are discontinued. In addition, because of the immunosuppressive effects of systemic therapy, it is crucial to continue treatment, while carefully managing the risk of potential side effects, such as infections caused by bacteria, fungi, or viruses. Therefore, there is a need to develop new treatment approaches that have as little systemic impact as possible.

Sphingolipids are one of the major lipid components of cell membranes and are involved in various physiological functions, such as the nervous, immune, and cardiovascular systems [6,7,8,9]. The sphingolipid metabolites ceramide, ceramide-1-phosphate (C1P), sphingosine, and sphingosine-1-phosphate (S1P) have been well studied in terms of regulating stress tolerance, proliferation, differentiation, and maturation in nervous system cells [10,11,12]. Therefore, it has been reported that sphingolipids are significantly involved in the pathogenesis of neurodegenerative diseases such as Parkinson’s disease, Alzheimer’s disease, and multiple sclerosis [13,14,15]. Moreover, ceramide and S1P have been shown to have roles in cell differentiation and proliferation [16,17]. Thus, several research groups have focused on understanding the impact of sphingolipids and the signaling pathways of S1P and its receptors on cancer biology [18].

In addition to these functions, sphingolipids are essential to maintaining the skin barrier function, the most critical component of protection for the human body [19,20]. Moreover, S1P–S1P-receptor (S1PR) signaling has also been reported to have a variety of roles in cell signaling and impacts inflammatory skin disorders such as atopic dermatitis, contact dermatitis, and psoriasis [21,22,23]. Immune cells, including dendritic cells and T lymphocytes, have long been focused on as the primary players in the pathogenesis of psoriasis. However, keratinocytes, located in the outermost layers of the skin, are the first cells to be stimulated and the last cells to cause epidermal thickening. In recent years, there has been a growing discussion about the potential role of sphingolipids—crucial molecules involved in skin barrier formation—and their metabolite S1P and S1PRs in the development of psoriasis [24]. This review focuses on the effects of sphingolipids, especially ceramide and S1P, and S1P–S1PR signaling on psoriasis.

## 2. Sphingolipid Metabolism

“Sphingolipids” is a generic term for lipids with a sphingoid base as their backbone, including sphingosine, ceramide, glycosphingolipids, and phosphosphingolipids. Phosphosphingolipids include S1P, C1P, and sphingomyelin [17,25,26,27]. It is believed that sphingomyelin serves as a reservoir molecule for ceramide. Ceramide is produced constantly and is used as an essential molecule in synthesizing complex sphingolipids and sphingomyelin, consisting of a sphingosine backbone that forms an amide bond with the carboxyl group of a fatty acid. In addition, there are three sphingolipid metabolic pathways in which ceramide is a crucial molecule: the de novo pathway, the salvage pathway, and the sphingomyelinase (SMase) pathway [28,29,30]. In the de novo pathway, which occurs on the cytosolic surface of the endoplasmic reticulum (ER) and other ER-like membranes, the biosynthesis of sphingolipids begins with the condensation reaction of serine and palmitoyl CoA catalyzed by serine palmitoyl transferase, followed by several reactions to form ceramide [31]. The de novo pathway synthesis consists of 3-ketosphinganine via the enzymatic condensation of serine and palmitoyl-CoA by serine palmitoyl transferase, which results in sphinganine production followed by 3-ketosphinganine reductase reduction. Then, sphinganine reacts with (dihydro) ceramide synthase (CerS; six mammalian CerS isoforms have been identified) to generate saturated ceramide precursor dihydroceramide. Lastly, the introduction of a 4, 5-trans double bond catalyzed by dihydroceramide desaturase produces ceramide [32]. In the salvage pathway, which occurs in late endosomes or lysosomes, sphingomyelin and complex glycosphingolipids are metabolized to ceramide, which is further converted to sphingosine via ceramidase catalysis. The generated sphingosine is salvaged for ceramide synthesis. In the SMase pathway, which occurs in the Golgi membrane or in the cell membrane, SMase breaks down sphingomyelin to synthesize the ceramide. This ceramide synthesis, which occurs via the direct hydrolysis of sphingomyelin by SMase, is reversible via the action of sphingomyelin synthase [33]. The synthesized ceramide then undergoes a cycle of synthesis and catabolism, returning to sphingomyelin through sphingomyelin synthase, forms C1P via ceramide kinase, and returns to glycosphingolipids via glycosylceramide synthase. Ceramide undergoes a process wherein it is converted into sphingosine by ceramidase. Sphingosine is then phosphorylated by two sphingosine kinases, SphK1 and SphK2, resulting in the formation of S1P. The S1P molecule has two possible outcomes: it can either enter the sphingolipid metabolic pool by being dephosphorylated by S1P phosphatase, or it can be broken down irreversibly into phosphoethanolamine and hexadecenal via S1P lyase, which depletes the pool. In addition, S1P can also function as a signaling molecule when transported extracellularly by S1P transporters [34,35].

As well as in physiological processes, sphingolipid metabolism, including neoplasm, is essential in pathological conditions [36]. Cancer is associated with alterations in enzyme activity, expression, and subcellular localization in sphingolipids, leading to apoptosis suppression and a diminished sensitivity to chemotherapeutic agents [37]. Thus, adjustments in sphingolipid metabolism facilitate innovative developmental approaches in designing antineoplastic pharmaceuticals [36]. In general, the sphingolipid mechanism nodes are divided into two main categories: one group promotes ceramide generation, and the other inhibits ceramide depletion. Hence, ceramide generation via de novo synthesis and sphingomyelin hydrolysis aids in treating cancer. On the other hand, the suppression of enzymes that deplete ceramide—for instance, ceramidases, glucosylceramide synthase, or SphKs—when combined with standard cancer therapies that dysregulate these enzymes, reveal promising improvements in the patient response to various cancer therapies [38,39,40].

## 3. Ceramide Roles in the Skin

The epidermis is made up of four layers, namely, the basal layer (stratum basale), the spinous layer (stratum spinosum), the granular layer (stratum granulosum), and the cornified layer (stratum corneum). Ceramides play fundamental roles in the stratum corneum, one of the critical components of the epidermal permeability barrier. Most ceramide synthesized in the epidermal granular layer is metabolized to glucosylceramide and sphingomyelin. The ceramides synthesized in the stratum corneum form lamellar structures with fatty acids and cholesterol to form the epidermal permeability barrier. The epidermis has a unique ceramide profile, and 21 ceramide subclasses have been identified in the human stratum corneum. The heterogeneity of these ceramide molecules is due to the sphingosine base variation in hydroxylation (non-hydroxy, 2-hydroxy, and terminal ω-hydroxy). In addition, there is also diversity in the chain lengths of the fatty acids and sphingosine bases. Most ceramides in the stratum corneum are non-hydroxy acyl sphingosine (NS) (carbon chain length: 16–24) [41,42,43]. The ceramide chain length is important to the stratum corneum structure and ceramide abundance, and short ceramide chains lead to a loss in barrier function [41,44,45,46]. Amide-linked fatty acids (carbon chain length and saturation) and heterogenous ceramides with appropriate ratios are also necessary for a vital barrier. Decreased acyl ceramide and non-hydroxy acyl 4-hydroxydihydrosphingosine (phytosphingosine), along with changes in a ceramide fatty acid chain length, affect lipid packing and the lamellar structure in the stratum corneum, causing the conditions found in several common skin diseases, including atopic dermatitis [20]. As epidermal ceramide is an important molecule for maintaining the skin barrier function, various ceramide-containing moisturizers have been developed, and their effects have been studied [47,48,49,50,51]. Hence, alterations to ceramides, in addition to their metabolites, can lead to a variety of skin diseases, such as atopic dermatitis, Netherton syndrome, psoriasis, and ichthyosis, in which the skin barrier function is greatly affected [52,53,54].

In addition to its role as a component of the skin barrier, ceramide has a role as a mediator lipid. Intracellular ceramide triggers apoptosis by inducing caspase-3 [55]. Environmental stimuli such as heat and oxidative stress activate SMase, which produces ceramide from sphingomyelin. Ceramide then activates caspases, which cause DNA degradation and cell nucleus destruction, resulting in apoptosis [56,57]. Several studies have reported that intracellular ceramide induces apoptosis by suppressing the PI3K/AKT signaling pathway [58,59,60]. However, some researchers have found that ceramide activates the PI3K/AKT pathway, and ceramide induces apoptosis only if the PI3K pathway is inhibited [61].

## 4. S1P and S1P–S1PR Signaling in the Skin

S1P is one of the sphingolipids produced via the degradation of ceramide and the phosphorylation of sphingosine, as described above. SphK, which produces S1P, and S1P lyase, a degrading enzyme, are expressed in most of the cells, and the production and degradation of S1P are constantly occurring [62]. Although S1P is produced intracellularly, it is then exported out of the cell via specific transporters [63] and acts as a signaling molecule through a family of G-protein-coupled receptors (S1PRs). In addition to extracellular signaling, S1P also functions intracellularly [64,65]. Intracellular S1P induces Ca^2+^ release from the ER, and activates MAPK and Rac1/IQGAP1 [66]. It has been theorized that the activation of SphK1 is a critical factor in the intracellular S1P signaling cascade [67]. In addition, S1P can activate the NF-κB pathway that regulates inflammation, immune responses, and cell survival. Based on previous studies, TNF-α provokes SphK activation, leading to S1P synthesis [68,69]. TNFα-induced NF-κB activation can be abrogated by the SphK inhibitor, N, N-dimethylsphingosine. TNF receptor-associated factor 2 (TRAF2), an essential ubiquitin E3 ligase for activating the canonical NF-κB pathway, directly interacts with SphK, which is crucial for TNF-induced NF-κB activation [68]. Moreover, S1P is a critical co-factor in the catalytic activity of TRAF2. The activation of TRAF2 by S1P produced by SphK on the plasma membrane connects sphingolipid metabolism to the NF-κB pathway [70].

Extracellular S1P binds to S1PRs on the plasma membrane and activates signaling that initiates cellular responses such as cell proliferation, differentiation, apoptosis, and immune responses [21]. Each of the five S1PRs is known to have a different effect on signaling pathways and cellular responses, and this response depends on the cell type in which the S1PRs are expressed [71]. S1PR1-3 is abundant in cardiovascular and immune system cells and is widely distributed in most other cell types. In contrast, S1PR4 and S1PR5 are expressed in limited cell types [72]. Regarding skin-resident cells, keratinocytes, the most critical cells covering the skin’s surface, have been reported to express all five receptors [73]. Neutrophils play an essential role in all inflammatory diseases, including infectious diseases and autoinflammatory diseases, preferentially expressing S1PR1 and S1PR4, with lower levels of S1PR3 and S1PR5 [74]. Basophils, which are involved in various allergic diseases, express S1PR1-4 [75]. Eosinophils express S1PR4 and S1PR5 [76]. Mast cells, which comprise one of the most critical cell types in allergic diseases, express S1PR1, S1PR2, and S1PR4, but not S1PR3 or S1PR5 [21]. Dendritic cells express all five S1PRs, but the gene expression levels vary according to the cell subtype [77,78]. Fibroblasts, an important component of the dermis, express S1PR1-3 predominantly [79].

According to previous reports, S1P–S1PR signaling pathways have crucial effects on the skin and on various skin diseases. In particular, S1PR1 and S1PR2 have been studied, and it has been reported that S1P–S1PR1 signaling increases TNF-α, IL-36γ, and IL-8 gene expression and IL-8 protein production in the keratinocytes [73]. The S1P–S1PR1 axis is also associated with atopic dermatitis, showing decreased S1PR1 protein expression in basophils in patients with atopic dermatitis [75]. In addition, S1PR1 signaling promotes angiogenesis during wound healing [80]. As the S1P–S1PR2 axis is essential to maintaining the skin barrier and protecting the skin from bacterial infection [81], the skin barrier is impaired in the S1PR2-deleted epidermis [23]. In contrast, S1PR2 blockage or deletion reduces atopic dermatitis and contact dermatitis inflammation [23,82]. This is thought to be because S1P–S1PR1 signaling is induced by allergens, bacteria, and other stimuli when S1PR2 is deficient [23], and S1P–S1PR1 signaling then leads to the recovery of tight junction protein expression [83].

## 5. Psoriasis

**Figure 1:**
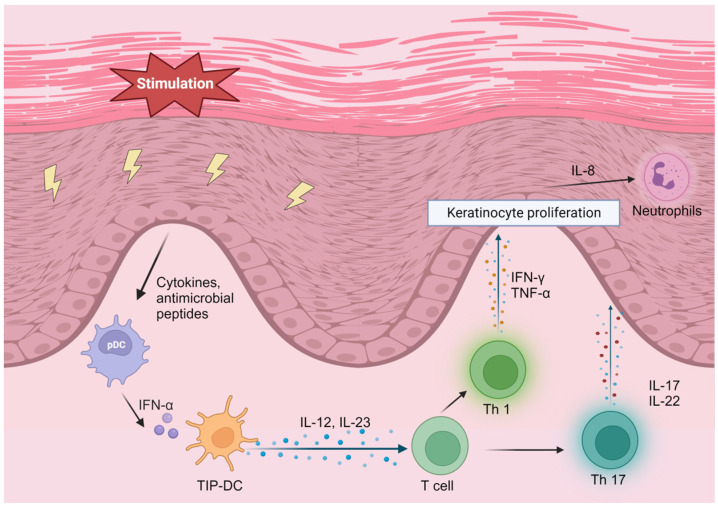
The psoriasis aggravation feedback loop. IFN, interferon; TNF, tumor necrosis factor; IL, interleukin; TIP-DC, TNF-α- inducible nitric oxide synthase-producing dendritic cell. Created using BioRender.com (www.biorender.com, accessed on 5 September 2023).

Psoriasis is a common chronic skin disorder characterized by erythematous plaques with silver scales. Psoriasis is categorized into several subtypes, including psoriasis vulgaris (plaque psoriasis), guttate psoriasis, pustular psoriasis, erythrodermic psoriasis, and psoriatic arthritis [1], with psoriasis vulgaris accounting for about 85–90% of psoriasis patients [2]. Approximately 50% of patients with psoriasis have itching, and 20–40% have nail lesions [84,85]. Although the prevalence of psoriasis varies by ethnicity, the age of onset is generally between adolescence and middle age [3]. In psoriasis lesions, numerous dendritic cells infiltrate the skin. Among the various types of dendritic cells, TNF-α-inducible nitric oxide synthase-producing dendritic cells (TIP-DCs) play an essential role in the pathogenesis of psoriasis. External stimulation activates keratinocytes to produce cytokines and antimicrobial peptides to induce TIP-DC-producing TNF-α, which increases the inflammatory response. TIP-DCs also produce IL-12 and IL-23 at the same time. IL-12 and IL-23 differentiate helper T cells into Th1 and Th17 cells. The inflammatory cytokines IL-17 and IL-22 are produced by Th17 cells, and interferon (IFN)-γ and TNF-α are produced by Th1 cells. These cytokines stimulate the inflammation and proliferation of epidermal keratinocytes, which induce inflammatory cytokine production from neutrophils, monocytes, and T lymphocytes. This negative loop of epidermal keratinocyte stimulation, TIP-DC activation, Th17 cell differentiation induction, and inflammatory cytokine production continually occurs in psoriasis lesions, contributing to the chronicity and severity of the disease [86] (Figure 1). Psoriasis can be managed through various treatment approaches. The two main approaches to treating psoriasis are reducing inflammation and reducing the over-proliferation of keratinocytes. Patients with mild-to-moderate psoriasis are treated with topical therapy, mainly with topical corticosteroids and vitamin D3 analogs [4,87]. Moderate-to-severe cases are treated with UV light therapy, systemic anti-inflammatory therapy, and biologic therapy [88]. Regarding biologics, several agents have been developed, including TNF-α inhibitors (Adalimumab, Etanercept, Infliximab, and Certolizumab pegol), as well as the IL-12/23p40 inhibitor (Ustekinumab), IL-17 inhibitors (Secukinumab, Brodalumab, and Ixekizumab,), and IL-23 inhibitors (Guselkumab, Tildrakizumab, Risankizumab, and Mirikizumab) [89]. In recent years, there has been increased attention on the JAK–signal-transducer-and-activator-of-transcription (STAT) pathway, which promotes inflammatory cytokine signaling and is also involved in the pathogenesis of psoriasis. This has led to the use of oral JAK inhibitors in the treatment of psoriasis [90,91]. JAK is an intracellular enzyme that binds to the cytoplasmic domain of cytokine receptors. When cytokines bind to the receptor, inflammatory signaling via JAK is initiated. When a cytokine binds to the receptor, a heterodimer of JAK is formed and autophosphorylated, and STAT is activated. The activated STAT then dimerizes and translocates to the cell nucleus, where it regulates gene transcription in various cytokines, including the proinflammatory cytokines that play a role in the pathogenesis of psoriasis [92]. JAK1, JAK2, and TYK2 are known to be involved in cell proliferation [93]. Currently, JAK1 inhibitors and TYK2 inhibitors are approved for psoriasis treatment [94]. As all systemic therapies, including biologic agents and JAK inhibitors, significantly affect the immune system, patients are exposed to various risks, such as bacterial, fungal, and viral infections, during treatment. Therefore, it is desirable to develop new treatment approaches that will impact the systemic immune system less.

The treatment of psoriasis has traditionally focused on targeting inflammatory cells such as lymphocytes and neutrophils. However, recent research has highlighted the role of keratinocytes, the outermost layer of skin cells, in the pathogenesis of psoriasis. Studies have shown that keratinocytes in psoriatic skin are hyperproliferative and produce large amounts of pro-inflammatory cytokines, activating immune cells and forming psoriatic plaques [95].

In particular, there is increasing evidence that the skin barrier function and lipid metabolism may be involved in the development of psoriasis. Disruption in the skin barrier function can lead to increased permeability and the exposure of the immune system to environmental triggers, leading to inflammation and psoriatic plaque formation. Abnormal lipid metabolism in the skin can also contribute to inflammation and keratinocyte hyperproliferation, further exacerbating psoriatic lesions [96].

These findings suggest that targeting the skin barrier function and lipid metabolism may constitute a potential new approach to the treatment of psoriasis. Further research is needed in order to fully understand the role of the skin barrier function and lipid metabolism in psoriasis and to develop effective treatments targeting these pathways.

## 6. Sphingolipids in Psoriasis

Psoriasis has a strong relationship with sphingolipids. Several groups have performed comparisons of the sphingolipid levels in healthy control skin, psoriatic lesional skin, and psoriatic non-lesional skin, and found an increase in sphingosine, S1P, and ceramide in psoriatic lesional skin [97,98]. Lipidomic profiling has also revealed that the skin lesions in human psoriasis patients and imiquimod (IMQ)-induced psoriasis mouse models have higher ceramides than those in the control group [99]. In contrast, Moskot et al. reported that the ceramide level in psoriatic keratinocytes was lower than in healthy control skin keratinocytes [100]. Nakajima et al. also reported the ceramide deficiency in the psoriasis patient epidermis and the psoriasiform epidermis in a mouse model [101].

Furthermore, the ceramide compositions in the stratum corneum, epidermal keratinocytes, and fibroblasts alter between patients with psoriasis skin and healthy control skin [102,103,104,105]. Tawada et al. used matrix-assisted laser desorption/ionization time-of-flight mass spectrometry (MALDI-TOF-MS) to analyze the ceramide profiles of the stratum corneum in healthy controls, atopic dermatitis, and psoriasis patients, showing a decrease in the molecular size of ceramides in the tape-stripped stratum corneum from psoriasis patients [106].

**Figure 2:**
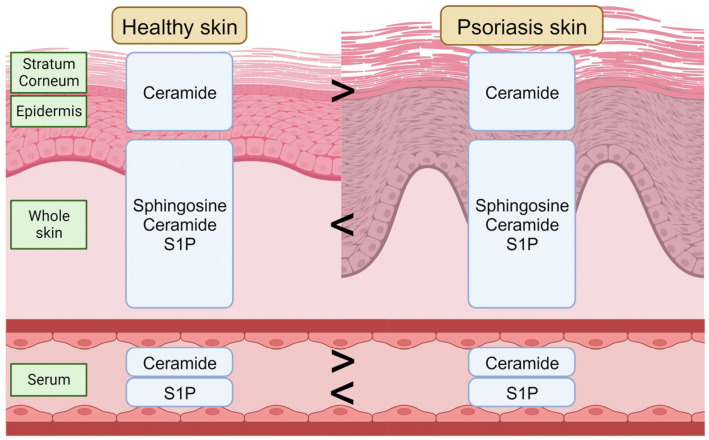
The sphingolipid composition in the healthy skin and in psoriasis skin. S1P, sphingosine-1-phosphate. Created using BioRender.com (accessed on 5 September 2023).

In addition to sphingolipids in the skin, serum sphingolipids have also been investigated with regard to the psoriasis mechanism. The ceramide composition in the serum is altered in psoriasis patients; the total ceramide level decreases, and the serum S1P level increases, in severe psoriasis patients [107,108]. A cross-sectional controlled study also showed an increase in the serum S1P level and a decrease in the serum ceramide level in psoriasis patients and a positive correlation between the psoriasis severity and serum S1P levels [109]. Moreover, the serum S1P level is lower in obese psoriasis patients (BMI > 30) compared to in normal-weight patients (BMI < 30) [110] (Figure 2). According to these studies, the application of ceramide, especially with a similar composition to healthy skin, might be a new approach to improving psoriasis skin conditions.

Although many studies have revealed an increased S1P level in psoriasis skin and serum, S1P has been reported to have protective effects in psoriasis [111]. Shaper et al. reported that topical S1P treatment significantly reduced ear swelling, reduced inflammatory cell infiltration, and reduced edema in the ear skin in an IMQ-induced psoriasis mouse model [111]. Moreover, Chen et al. showed a mouse model that developed psoriasis-like skin lesions due to S1P dysfunction [112]. These studies suggest new approaches around the theory that the administration of S1P may be a potential target for psoriasis treatment. However, it seems inconsistent that S1P administration improves psoriasis symptoms while S1P levels are elevated in psoriasis. Regarding the reason behind this inconsistency, S1PR signaling is likely to have an effect, as each S1PR has a different role in cell function, as described in the previous section.

## 7. S1P–S1PRs in Psoriasis

The relationship between S1PR and psoriasis has been studied extensively. A recent study has shown that S1PR1-5 protein expression is increased in the skin of an IMQ-induced psoriasis mouse model and that S1PR1-5 expression is reduced after treatment [113]. In addition, a GWAS study showed that only S1PR1 gene expression decreases in the lesional skin of psoriasis patients compared to non-lesional skin, with no change in the S1PR2-5 gene expression [114]. It is worth noting that S1PR is not only associated with psoriasis and healthy individuals but also impacts both lesional and non-lesional areas of psoriasis skin.

Among the five S1PRs, S1PR1 in particular has been studied primarily with regard to its relevance to psoriasis. In the IMQ-induced mouse psoriasis model, myeloid S1PR1 deletion leads to increased psoriasis inflammation through increased blood vessels [114]. In addition, several studies have recently revealed that the S1PR1 modulator ameliorates psoriasis. Ji et al. treated psoriasis animal models (sodium-lauryl-sulfate-induced mouse skin irritation model, diethylstilbestrol-induced mouse psoriasis model, and propranolol-induced guinea pig psoriasis model) with the selective S1PR1 modulator Syl930, and found that Syl930 ameliorates psoriasis [115]. Vaclavkova et al. reported a phase-II clinical trial in plaque psoriasis, showing that the oral S1PR1 agonist ponesimod improved psoriasis, with at least a 75% reduction. However, the trial did not proceed to phase III due to severe adverse effects [116]. In addition, a case was reported in which a patient developed psoriasis while receiving the S1PR1 modulator fingolimod for multiple sclerosis (MS) and was treated with the anti-IL-17 treatment secukinumab [117]. Moreover, a case of generalized pustular psoriasis was also recently reported after the patient had received fingolimod for the treatment of MS [118]. Thus, as oral S1PR1 modulators cause severe side effects or paradoxical effects to increase neutrophil activity [119], the development of topical S1PR1 modulators has been encouraged [120].

Although there has been little research on the mechanism by which S1PRs other than S1PR1 affect psoriasis, it has been reported that S1PR2 gene expression is elevated in the skin of mice in which a Western diet activates the Th17 pathway [121]. Furthermore, the relationship between S1PR3 and psoriasis has also been studied to some extent, and RT-qPCR and immunohistochemistry have shown that the S1PR3 gene and protein expression are downregulated in human psoriasis skin [122]. This is believed to be because S1PR3 affects cell proliferation and inflammation in psoriasis via the AKT/mTOR pathway [122]. As S1PR4 signaling is known to regulate dendritic cell function and Th17 T-cell differentiation [123], it has been studied for possible involvement in the pathogenesis of psoriasis. Dillmann et al. reported that S1PR4 signaling attenuates TLR-induced IFN-α production via plasmacytoid dendritic cells [124]. Moreover, Schuster et al. found that S1PR4 deletion does not induce IL-17 alteration but reduces macrophage infiltration and CCL2 production [125]. Although it remains to be examined directly whether S1PR5 affects the pathogenesis of psoriasis, there are several reports of S1PR modulators also acting on S1PR5, influencing psoriasis [126,127].

## 8. S1PR Modulators for Psoriasis

The first S1PR modulator, fingolimod, was developed and was FDA approved in 2010 for multiple sclerosis (MS) treatment [128]. Fingolimod is a selective S1PR1 agonist but also binds to S1PR3-5. Since then, several S1PR modulators have been developed, and clinical trials have been performed in MS, inflammatory bowel disease (IBD, particularly ulcerative colitis (UC)), atopic dermatitis, graft-versus-host disease (GVHD), and psoriasis [129,130,131,132]. Siponimod, a selective S1PR1 and S1PR5 modulator, acts as a functional antagonist to induce S1PR1 internalization and degradation in T and B cells. Siponimod was FDA-approved for oral treatment for MS in 2019. Ozanimod, a selective S1PR1 and S1PR5 modulator, was FDA approved for MS in 2020 and for UC in 2021 [133]. Ponesimod, a selective S1PR1 modulator, was FDA approved for MS in 2021. Etrasimod, a selective S1PR1, S1PR4, and S1PR5 modulator, has been investigated in UC [129]. Although several S1PR modulators are in development and have already been approved for autoimmune diseases such as MS, and IBDs such as UC, there is no approved S1PR modulator for psoriasis yet [132]. However, as mentioned in the previous section, several reports show evidence that S1PRs are involved in the pathogenesis of psoriasis, and meta-analyses have been conducted to confirm these studies [134]. Therefore, it is desirable to attempt clinical trials of other S1PR modulators for psoriasis patients or to develop S1PR modulators that can be administered topically, rather than systemically, to minimize systemic side effects.

## 9. Conclusions

Psoriasis is a complex skin condition that has been extensively studied with the focus on immune cells, such as dendritic cells and lymphocytes. However, recent research has shown that keratinocytes, the primary cells in the epidermis, also play a significant role in the pathogenesis of psoriasis. It has been discovered that sphingolipids, essential to the barrier function of the epidermis, are involved in the cells’ immune response. This new insight could pave the way to the development of novel therapeutic approaches to treating psoriasis by targeting sphingolipids and their metabolites.

## Data Availability

Not applicable.
